# Kaempferol Improves Exercise Performance by Regulating Glucose Uptake, Mitochondrial Biogenesis, and Protein Synthesis via PI3K/AKT and MAPK Signaling Pathways

**DOI:** 10.3390/foods13071068

**Published:** 2024-03-30

**Authors:** Xiaoning Ji, Chaozheng Zhang, Jing Yang, Yaru Tian, Lijuan You, Hui Yang, Yongning Li, Haibo Liu, Deng Pan, Zhaoping Liu

**Affiliations:** 1 State Key Laboratory for Quality Ensurance and Sustainable Use of Dao-di Herbs, National Resource Center for Chinese Materia Medica, China Academy of Chinese Medical Sciences, Beijing 100700, China; 2 NHC Key Laboratory of Food Safety Risk Assessment, China National Center for Food Safety Risk Assessment, Beijing 100022, Chinayanghui@cfsa.net.cn (H.Y.); liuzhaoping@cfsa.net.cn (Z.L.)

**Keywords:** kaempferol, exercise performance, mitochondria function, glucose uptake, protein synthesis

## Abstract

Kaempferol is a natural flavonoid with reported bioactivities found in many fruits, vegetables, and medicinal herbs. However, its effects on exercise performance and muscle metabolism remain inconclusive. The present study investigated kaempferol’s effects on improving exercise performance and potential mechanisms in vivo and in vitro. The grip strength, exhaustive running time, and distance of mice were increased in the high-dose kaempferol group (*p* < 0.01). Also, kaempferol reduced fatigue-related biochemical markers and increased the activities of superoxide dismutase (SOD) and glutathione peroxidase (GSH-Px) related to antioxidant capacity. Kaempferol also increased the glycogen and adenosine triphosphate (ATP) content in the liver and skeletal muscle, as well as glucose in the blood. In vitro, kaempferol promoted glucose uptake, protein synthesis, and mitochondrial function and decreased oxidative stress in both 2D and 3D C2C12 myotube cultures. Moreover, kaempferol activated the PI3K/AKT and MAPK signaling pathways in the C2C12 cells. It also upregulated the key targets of glucose uptake, mitochondrial function, and protein synthesis. These findings suggest that kaempferol improves exercise performance and alleviates physical fatigue by increasing glucose uptake, mitochondrial biogenesis, and protein synthesis and by decreasing ROS. Kaempferol’s molecular mechanism may be related to the regulation of the PI3K/AKT and MAPK signaling pathways.

## 1. Introduction

With the escalating global popularity of recreational sports and fitness culture, improving exercise capacity safely and legally has become an increasingly crucial research goal [1]. A decline in exercise capacity and premature exercise-induced fatigue are closely associated with the function of skeletal muscles [2]. Energy deficiency emerges as the primary contributor, where the depletion of glycogen and glucose results in insufficient adenosine triphosphate (ATP) production and compromised contractile function. Enhancing mitochondrial biogenesis and oxidative phosphorylation increases ATP production in response to the increased energy demands that occur during exercise, whereas promoting glycogen synthesis and glucose uptake helps maintain intracellular energy stores for prolonged contraction [3]. The accumulation of reactive oxygen species (ROS) produced by the mitochondrial respiratory chain also causes fatigue, decreasing exercise performance [4]. Conversely, it is of paramount importance to ensure the effective disposal of metabolic byproducts, such as lactic acid (LA) and blood urea nitrogen (BUN), whose accumulation could detrimentally affect muscular functions and the overall operational efficiency [5,6,7].

Skeletal muscle is a key tissue for movement, metabolic homeostasis, and energy expenditure in the body; its precise regulation is crucial for maintaining body function, especially for exercise performance [8]. The functioning and strength of skeletal muscle are vastly influenced by glucose intake, protein synthesis, mitochondrial functionality, and antioxidant capacity [9,10,11]. Cellular signaling pathways, such as PI3K/AKT and MAPK cascades, are crucial regulators of the growth, metabolism, and function of skeletal muscle cells [12,13]. PGC-1α functions as a downstream factor of these metabolic pathways, where it binds to and regulates several transcription factors, such as glucose transporter-4 (GLUT4) and mitochondrial transcription factor A (mtTFA), thereby enhancing mitochondrial respiration, promoting mitochondrial biogenesis and glucose uptake, and improving energy metabolism [14,15]. A study reported that the PI3K/AKT pathway stimulates protein synthesis and induces muscle growth via rapamycin complex (mTOR) kinase, thereby activating ribosomal protein S6 kinase (p70S6K) and 4E-binding protein-1 (4EBP1), a critical intracellular pathway involved in regulating muscle protein synthesis [16,17]. In some cases, athletes use prohibited substances, including stimulants, β-blockers, and anabolic–androgenic steroids, which violate anti-doping policies and expose athletes to the risks of adverse cardiovascular, neurological, endocrine, and other side effects [18]. Therefore, several recreational and elite athletes are incorporating traditional/natural medicinal and food components, such as caffeine, creatine, *Chinese ginseng*, nitrates, and polyphenols, into their exercise regimens [19,20,21]. Kaempferol is a natural flavonoid present in many fruits, vegetables, and medicinal herbs, including broccoli, cabbage, beans, leek, tomato, strawberries, grapes, and propolis [22]. Average intakes of individual kaempferol among US adults and Chinese adults are 5.4 mg/day and 9.3 mg/day, respectively [23,24]. The oral bioavailability is approximately 1.5% based on urinary excretion [25]. Additionally, research into kaempferol’s metabolism and pharmacokinetics in the body indicate that orally administered kaempferol undergoes extensive metabolism in the small intestine epithelial cells, liver, and other tissues, generating various metabolites, such as kaempferol sulfates and kaempferol glucuronides [26]. It has been studied extensively for antioxidant, anti-inflammatory, anticancer, and cardioprotective effects, which are highly relevant to exercise performance and recovery [25]. Specifically, its antioxidant property may help counteract oxidative damage induced by intensive training or competitions, thereby ameliorating muscle injury and preserving muscle function [27]. Studies have suggested that kaempferol may promote glucose uptake, thereby augmenting the availability of a substrate for energy production during exercises [28]. However, its specific effects and molecular mechanisms underlying exercise performance are not yet fully understood.

We hypothesize that kaempferol can enhance exercise performance and alleviate exercise-induced fatigue by regulating energy metabolism, protein synthesis, and antioxidant capacity. Therefore, the present study investigated the effect and potential mechanism by which kaempferol improves exercise performance in vivo and in vitro. We assessed the effects of kaempferol on grip strength and exercise capacity in C57BL/6 mice. Furthermore, we demonstrated the effects of kaempferol on promoting glucose uptake, alleviating oxidative stress, and enhancing mitochondrial biogenesis using both 2D and 3D cultures of C2C12 myotubes in vitro. Transcriptomic sequencing, immunofluorescence, and a high-content imaging analysis were conducted to identify potential mechanisms underlying kaempferol’s effects on C2C12 cells. Kaempferol might be a useful nutrient supplement to alleviate exercise-induced fatigue and enhance exercise performance.

## 2. Materials and Methods

### 2.1. Materials

The kaempferol (purity > 97%, CAS 520-18-3) was obtained from Mackin Regent (Shanghai, China) and stored at room temperature in the dark. The Dulbecco’s Modified Eagle’s Medium (DMEM), fetal bovine serum (FBS), horse serum, phosphate-buffered saline (PBS, pH 7.4), and penicillin-streptomycin (P/S) were acquired from Gibco (New York, NY, USA). The Cell Counting Kit-8 (CCK-8) was provided by Dojindo (Kumamoto, Japan). The MitoTracker Deep Red and 2-[N-(7-nitrobenz-2-oxa-1,3-diazol-4-yl)amino]-2-deoxy-Dglucose (2-NBDG) were obtained from Invitrogen (Carlsbad, CA, USA). The puromycin was generated by MCE (Shanghai, China). The Cell Staining Buffer was bought from Biolegend (San Diego, CA, USA). The primary antibodies were p-p38MAPK (Thr180/Tyr182, #4511, 1:1000), p-JNK (Thr183/Tyr185, #9255, 1:200), p-ERK (Thr202/Tyr204, #4370, 1:200), p-mTOR (Ser2448, #5536, 1:100), and p-AKT (Ser473, #4060, 1:200); these antibodies were sourced from Cell Signaling Technology (Beverly, MA, USA). p-P13K (AF3242, 1:200) and p-4EBP1 (AF3830, 1:200) were procured from Affinity Biosciences (Liyang, China). mtTFA (ab252432, 1:200) and GLUT4 (ab33780, 1:200) were purchased from Abcam (Cambridge, MA, USA). PGC-1α (ST1202) and puromycin (MABE343) were provided by Merck (Billerica, MA, USA). p-p70S6 K1 (Thr389, AP0564) was obtained from Abclonal Technology (Wuhan, China). The anti-mouse IgG (1:1000) and anti-rabbit IgG (1:1000) were sourced from Cell Signaling Technology (Beverly, MA, USA). The assay kits used for ATP (A095-1–1), glycogen (A043-1-1), malondialdehyde (MDA), superoxide dismutase (SOD), and glutathione peroxidase (GSH-Px) were purchased from the Nanjing Jiancheng Bioengineering Institute (Nanjing, China).

Seven-week-old special pathogen-free male C57BL/6 mice were obtained from Beijing Vital River Laboratory Animal Technology Co., Ltd. (Beijing, China). The C2C12 mouse skeletal myoblasts were obtained from Pythonbio (Guangzhou, China). The grip tester (DS2-20N) was obtained from Jiangsu Science Biological Technology Co., Ltd. (Nanjing, China). A motorized treadmill was provided (ZH-PT/5S) by Anhui Zhenghua Biologic Apparatus Facilities Co., Ltd. (Huaibei, China). The black-bottom 96-well plates and 96-well round-bottomed plates were purposed from Corning (Kennebunk, ME, USA). The RNA Nano 6000 Assay Kit of the Bioanalyzer 2100 system was obtained from Agilent Technologies (Santa Clara, CA, USA).

### 2.2. Animals and Treatment

In caring for and treating the experimental mice, we adhered to the ethical guidelines for the humane treatment of animals used in laboratory research. The seven-week-old special pathogen-free male C57BL/6 mice were obtained from Beijing Vital River Laboratory Animal Technology Co., Ltd. (Beijing, China). The mice were maintained at a constant temperature (22 ± 1 °C), humidity (55 ± 10%), and 12 h light/dark cycle; they had free access to water and food. After a 1-week adaptation period, the mice were randomly divided into three groups (*n* = 12): control (CT), low-dose kaempferol (KL), and high-dose kaempferol (KH). The CT group was administered with the solvent carboxymethyl cellulose sodium (CMC, 0.5% wt./vol), the KL group was gavagely administered with kaempferol (25 mg/kg bw [bw, body weight]), and the KH group was also administrated with kaempferol (100 mg/kg bw) as previously described [29,30]. A gavage solution was prepared by mixing kaempferol with 0.5% (wt./vol) CMC. The mice were gavaged daily with 0.2 mL/10 g bw/d.

**Figure 1:**
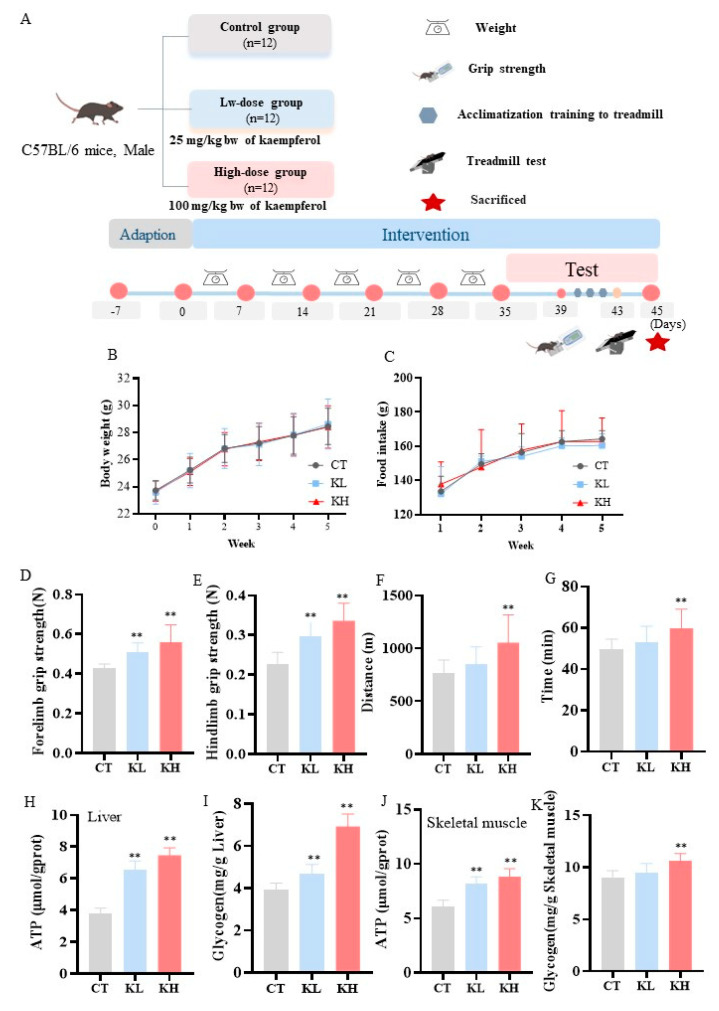
Effect of kaempferol on body weight, food intake, grip strength, exercise capacity, ATP, and glycogen in mice. (**A**) Experimental study design; (**B**) body weight; (**C**) food intake; (**D**) forelimb grip strength; (**E**) hindlimb grip strength; distance (**F**) and duration (**G**) of exercise until exhaustion; ATP (**H**) and glycogen (**I**) in the liver; and ATP (**J**) and glycogen (**K**) in skeletal muscle. CT, control group, 0.5% (wt./vol) solvent carboxymethyl cellulose sodium (CMC); KL, 25 mg/kg bw of kaempferol; KH, 100 mg/kg bw of kaempferol; and bw, body weight. **, *p* < 0.01.

At the end of the experiment, the whole blood samples were gathered in the right eyes of the mice. Then, the mice were sacrificed by cervical dislocation. The liver and hindlimb skeletal muscle tissues of the mice were immediately dissected, frozen in liquid nitrogen, and kept at −80 °C. All the animal experiments were performed according to the procedures approved by the Institutional Animal Care and Use Committee of China National Center for Food Safety Risk Assessment (Approval no. 2023072). The animal experiments were performed according to the chart displayed in Figure 1A.

### 2.3. Grip Strength

The grip strength was measured in the mice. A grip tester was used to evaluate the forelimb and hindlimb grip strength of the mice. The mice were positioned horizontally on the wire grid so that their forelimbs grasped the grid. After the mice had grasped the grid with their forelimbs, their tails were pulled backward evenly along the horizontal plane with increasing force until they released the grid. The peak force during this process was recorded using the instrument when the mice had released the grid. The measurements were repeated six times for each mouse and averaged to obtain the grip strength value, which was used to evaluate the forelimb grip strength [31]. The hindlimb grip strength in the mice was performed similarly to the aforementioned forelimb evaluation. Briefly, the mice’s hindlimbs grasped the metal grid and, until they released their grip, were pulled backward evenly with increasing force. A series of six trials were conducted on each mouse, and the average of the peak force measurements was calculated.

### 2.4. Exercise Capacity

Prior to exercise, the mice were acclimated to a motorized treadmill for 3 days (day 1: running 10 m/min at a slope of 0 for 10 min; day 2: running 10 m/min at a slope of 0 for 5 min and 10 m/min at a slope of 5° for 5 min; and day 3: running 10 m/min for 5 min and 15 m/min at a slope of 10° for 5 min). To access exercise capacity, we placed the mice on the treadmill and they warmed up with a slope of 5° for 5 min, followed by a slope of 10° for 5 min at 10 m/min. The initial speed was 10 m/min for 5 min at a slope of 10° and then increased (2 m/min every 5 min) up to 34 m/min, referring to previous studies [14]. Exhaustion was defined as receiving > 7 shocks within 15 s. Finally, the exercise time and distance to exhaustion were recorded.

### 2.5. Measurement of Tissue Glycogen and ATP

The ATP and glycogen levels in the liver and skeletal muscle were determined using commercial assay kits according to the manufacturer’s instructions.

### 2.6. Blood Biochemical Assessments

The whole blood glucose levels were tested using the Accu-Check Active Blood Glucose Meter (Roche, Shanghai, China). The serum was promptly centrifuged at 3500 rpm for 10 min at 4 °C and then stored at 4 °C. The levels of lactic acid (LA), lactate dehydrogenase (LDH), BUN, creatine kinase (CK), triglyceride (TG), total cholesterol (TC), alanine aminotransferase (ALT), and aspartate aminotransferase (AST) were determined using an auto-analyzer (AU680, Beckman Coulter, Brea, CA, USA). The levels of MDA, SOD, and GSH-Px were determined using a commercially available enzyme-linked immunosorbent assay (ELISA) kit according to the manufacturer’s protocols.

### 2.7. 2D/3D Cells Culture and Treatment

The C2C12 mouse skeletal myoblasts were obtained from Pythonbio (Guangzhou, China). The cells were grown in DMEM supplemented with 10% FBS and 1% P/S. At 70–80% confluence, the cells were cultured in DMEM supplemented with 2% horse serum and 1% penicillin-streptomycin for six days to induce differentiation into the myotubes [32]. All the cells were maintained at 37 °C in a humidified atmosphere containing 5% CO_2_. The culture media were replaced every two days.

After differentiation, the cells were seeded at 1 × 10^4^ cells per well into black-bottom 96-well plates (Corning, Kennebunk, ME, USA) overnight for the 2D culture. The cells were seeded at 2000–3000 cells/well into ultra-low attachment 96-well round-bottomed plates (Corning, Kennebunk, ME, USA) and then cultured for three days to generate skeletal muscle spheroids (3D culture) [33]. The cells were then treated with varying concentrations of kaempferol (50, 25, 12.5, and 6.25 μM) for 24 h in the 2D culture and 48 h in the 3D culture. The treatment concentration of kaempferol was determined based on our previous cell activity experiments. The cells were cultured at 37 °C in a humidified atmosphere containing 5% CO_2_. The testing was subsequently commenced.

### 2.8. Transcriptome Sequencing

The C2C12 myotubes were cultured with 25 μM kaempferol for 24 h. The total RNA was extracted from the cells with TRIzol. RNA sequencing was performed by Novogene Biotech Co., Ltd. (Beijing, China). The RNA integrity was assessed using the RNA Nano 6000 Assay Kit of the Bioanalyzer 2100 system (Agilent Technologies, Santa Clara, CA, USA). A differential expression analysis of the groups was performed using the R package “DESeq2” (1.20.0). Differentially expressed genes (DEGs) were identified using DESeq2 with *p* < 0.05 and |log_2_(Fold Change)| > 0 [34,35]. A Kyoto Encyclopedia of Genes and Genomes (KEGG) pathway analysis of the DEGs was performed using the R package “cluster Profiler”. A gene set enrichment analysis (GSEA) was performed locally using the latest version of the GSEA analysis tool (http://www.broadinstitute.org/gsea/index.jsp, accessed on 22 December 2022). The raw RNA sequencing data are available through the National Center for Biotechnology Information Gene Expression Omnibus (NCBI–GEO) database (http://www.ncbi.nlm.nih.gov/geo/, accessed on 2 February 2024) under the following accession number: GSE252790.

### 2.9. Immunofluorescence Staining and Cell High-Content Imaging Analysis

#### 2.9.1. Glucose Uptake

The C2C12 myotubes in glucose-free DMEM were incubated for 4 h. After removing the culture medium, the cells were washed twice with PBS. The cells were then treated with 2-NBDG at 400 μM at 37 °C for 30 min, protected from the light.

#### 2.9.2. Measurement of ROS

The ROS levels were determined using a fluorescent probe (CellROX Green reagent, ThermoFisher Science, Carlsbad, CA, USA). To induce ROS, we cultured the cells with 100 μM tert-butyl hydroperoxide (TBHP) for 30 min. The cells were then stained with the CellROX Green reagent for 30 min at 37 °C, protected from the light.

#### 2.9.3. Measurement of Mitochondrial Mass and Mitochondrial Membrane Potential

The mitochondrial membrane potential and mitochondrial mass were measured using the TMRM reagent (Thermo Scientific, Carlsbad, CA, USA) and MitoTracker (Thermo Scientific, Carlsbad, CA, USA), respectively, according to the manufacturer’s instructions. The cells were washed twice with PBS and then loaded with TMRM or MitoTracker at 37 °C for 30 min, protected from the light.

#### 2.9.4. Protein Synthesis Measurements

Protein synthesis was measured in vitro in the C2C12 myotubes using the SUnSET method. Briefly, the C2C12 cells were incubated with 1 µM puromycin for the last 60 min of the experimental treatments. The puromycin expression was analyzed as described below.

#### 2.9.5. Expression of Key Target Proteins

The C2C12 cells were fixed with fixative buffer (eBioscience Fixation:Fixation/Permeabilization, 1:3 ratio) from the eBioscience Foxp3 Kit (ThermoFisher Science) at room temperature for 30 min. The cells were then washed twice with the permeabilization buffer and then incubated with primary antibodies (puromycin, p-p38 MAPK, ERK1/2, p-JNK, p-AMPK, p-mTOR, p-p70S6K, p-4EBP1, GLUT4, PGC-1α, and mtTFA) at room temperature for 30 min. After washing twice, the cells were probed using anti-mouse IgG or anti-rabbit IgG (1:1000, CST) for 1 h at room temperature.

#### 2.9.6. High-Content Imaging Analysis

The cells were washed twice with PBS or Cell Staining Buffer (Biolegend, San Diego, CA, USA). Finally, the cells were counterstained for nuclei with Hoechst 33342 (Solarbio, Beijing, China) and analyzed using Image Xpress software (Version 6.5, Molecular Device, LLC, San Jose, CA, USA) [36].

### 2.10. Statistical Analyses

The data analyses were performed using GraphPad Prism 8.3 (GraphPad Software Inc., San Diego, CA, USA). The data are expressed as the mean ± standard deviation (SD). A statistical analysis was performed using the one-way analysis of variants (ANOVA), followed by Fisher’s least significant difference (LSD) multiple comparisons. The level of statistical significance was set at *p* < 0.05.

## 3. Results and Discussion

### 3.1. Effect of Kaempferol on Grip Strength and Exercise Capacity

The body weights and food intake of the mice involved in the 35 days before the behavioral experiment were measured and recorded (Figure 1B,C). There was no significant difference in the body weight and food intake between the CT, KL, and KH groups. Continuous kaempferol supplementation did not influence the body weight and food intake when compared with the CT group.

Grip strength, exhaustive running time, and distance are important parameters for assessing muscle strength and exercise capacity in mice. The grip strength testing was performed first (Figure 1D,E); the KL and KH groups significantly increased the forelimb grip strength by 18.0% and 30.1%, respectively, compared with the CT group. Kaempferol at doses of 25 and 100 mg/kg bw significantly increased the hindlimb grip strength by 30.0% and 47.7%, respectively. Compared with the control CT group, kaempferol prolonged the exhaustion distance in the mice, with the KH group intensifying by 37.3% (*p* < 0.01) (Figure 1F). Similarly, the exhaustion time was significantly increased by 21.1% in the KH group (*p* < 0.01) (Figure 1G). These results indicate that kaempferol could substantially improve muscle strength and enhance the exercise capacity of mice, especially in the KH group.

### 3.2. Effect of Kaempferol on the ATP and Glycogen Contents in the Liver and Skeletal Muscle

The concentrations of ATP and glycogen—important energy sources during exercise and indicators of exercise-induced fatigue—in the liver and skeletal muscle were measured. The ATP contents of the liver in the KH (7.46 μmol/g prot) and KL (6.54 μmol/g prot) groups were significantly increased by 1.99-fold and 1.74-fold, respectively, compared with that in the CT group (3.75 μmol/g prot) (Figure 1H, *p* < 0.01). Furthermore, the glycogen level in the KH group increased by 75% from 3.94 ± 0.29 to 6.92 ± 0.56 mg/g (*p* < 0.01) compared with the CT group (Figure 1I). Similarly, as the kaempferol dose increased, the ATP and glycogen content in the skeletal muscle also tended to increase (Figure 1J,K). Kaempferol can increase the ATP and glycogen levels in the liver and skeletal muscle, which might provide or supplement sufficient energy to meet energy demands during exercise and promote physical recovery after exercise [5].

### 3.3. Effect of Kaempferol on Biochemical Parameters after Exercise

**Figure 2:**
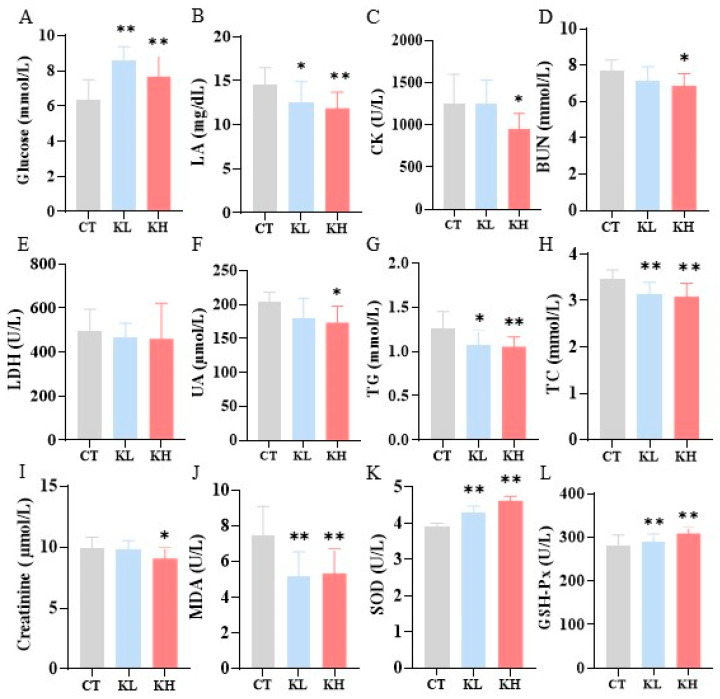
Effect of kaempferol on serum biochemical parameters in mice. (**A**) glucose; (**B**), lactic acid (LA); (**C**), creatine kinase (CK); (**D**), blood urea nitrogen (BUN); (**E**), lactate dehydrogenase (LDH); (**F**), uric acid (UA); (**G**), triglyceride (TG); (**H**), total cholesterol (TC); (**I**), creatinine; (**J**), malondialdehyde (MDA); (**K**), superoxide dismutase (SOD); (**L**), glutathione peroxidase (GSH-Px). CT, control group, 0.5% (wt./vol) solvent carboxymethyl cellulose sodium (CMC); KL, 25 mg/kg bw of kaempferol; KH, 100 mg/kg bw of kaempferol; bw, body weight. *, *p* < 0.05; **, *p* < 0.01.

Figure 2 depicts the effect of kaempferol on fatigue-associated biochemical markers, such as glucose, BUN, LA, CK, and LDH, after exercise [5]. Kaempferol significantly increased the glucose levels in the blood compared with the CT group (*p* < 0.01). By contrast, kaempferol significantly and dose-dependently decreased the LA levels (14.20–18.51%) compared with the CT group. Glucose is an important fuel for contracting muscle. Kaempferol may increase blood glucose levels because of its ability to promote glycogenolysis and release glucose into the bloodstream, providing an energy substrate for exercise [37]. The dose-dependent reduction in LA suggests that kaempferol may mitigate lactic acidosis-induced muscular fatigue during exercise by facilitating lactate clearance. Compared with the CT group, the CK level was significantly decreased in the KH group (*p* < 0.05) but not in the KL group. CK is an indicator for evaluating the degree of muscle damage. The decrease in CK indicates that kaempferol may have some muscle protection effects, which can alleviate exercise-induced muscle injury, thereby enhancing exercise capability. Moreover, the BUN level was significantly decreased in the KH group (*p* < 0.05) but not in the KL group.

Figure 2 presents the biochemical parameters related to liver, kidney, heart, and muscle injury markers. There was no significant difference in the levels of AST and ALT (both liver makers) among the three groups (Figure S1). The UA and creatinine—important indicators for evaluating kidney function—were significantly decreased in the KH group compared with the CT group. An increase in BUN and UA levels during exercise leads to a decline in performance and fatigue [7]. The decrease in UA, creatinine, and BUN implies improved kidney function in the KH group, which may facilitate the clearance of exercise-induced metabolites. The kaempferol significantly decreased the TG and TC levels in a dose-dependent manner. Studies have demonstrated that lipid profiles are associated with fatigue and reduced exercise tolerance [38].

Excessive oxidative stress triggering plays a crucial role in contributing to exercise-induced fatigue, potentially reducing exercise performance [4]. MDA is considered a presumptive biomarker for lipid peroxidation, whereas SOD and GSH-Px are important antioxidants in oxidative stress systems. Serum MDA concentrations of the KH and KL groups were significantly lower (*p* < 0.01) than that of the CT group (Figure 2J–L). Kaempferol accelerated the SOD and GSH-Px activities in the serum, especially in the KH group (all *p* < 0.01), compared with the control. Kaempferol effectively reduced the serum MDA levels, indicating a decrease in lipid peroxidation. This finding aligns with previous research that excessive oxidative stress contributes to exercise-induced fatigue and may impair exercise performance [39]. Therefore, kaempferol possesses antioxidation capacity and positively modulates the antioxidant defense system, thereby improving exercise performance.

In summary, kaempferol enhances the grip strength and exercise capacity in mice by increasing the forelimb and hindlimb grip strength, as well as prolonging the exhaustive running distance and time. Additionally, kaempferol increases the ATP and glycogen contents in the liver and skeletal muscle, providing more energy during exercise and promoting physical recovery after exercise. Kaempferol also has a positive effect on fatigue-associated biochemical markers, such as glucose, LA, and CK, as well as liver, kidney, and heart function markers, and it also decreases the accumulation of metabolic byproducts. Furthermore, kaempferol reduces oxidative stress and increases antioxidant activity, thereby improving exercise performance.

### 3.4. Effect of Kaempferol on the Glucose Uptake, Protein Synthesis, and Mitochondrial Biogenesis in Both 2D and 3D C2C12 Myotube Cultures

**Figure 3:**
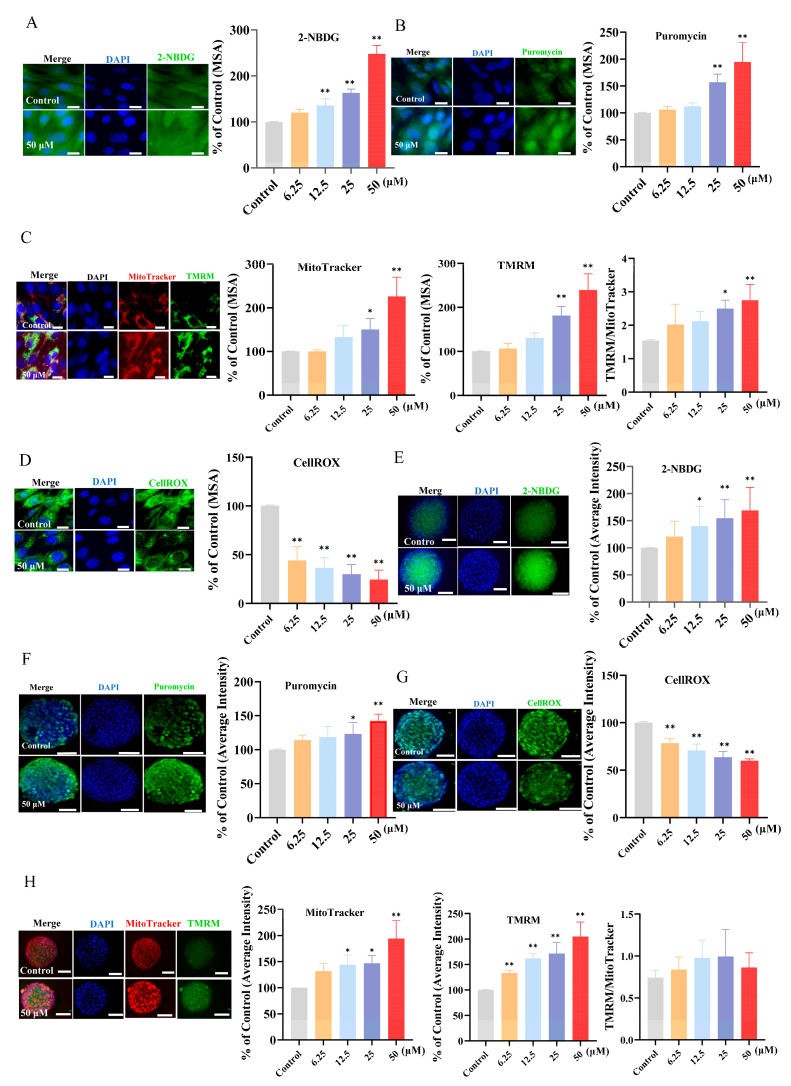
Effect of kaempferol on glucose uptake, mitochondrial mass, mitochondrial membrane potential, reactive oxygen species (ROS), and protein synthesis in vitro (C2C12 myotubes, cultured in 2D and 3D). The mean stain area (MSA) of 2-NBDG (**A**), puromycin (**B**), TMRM and MitoTracker (**C**), and CellROX (**D**) in C2C12 myotubes cultured in 2D. The average intensity of 2-NBDG (**E**), puromycin (**F**), CellROX (**G**), and TMRM and MitoTracker (**H**) in C2C12 myotubes cultured in 3D. Left panel: representative images, bar size: 50 μm (2D) and 100 μm (3D). The assays were performed in triplicate with three technical replicates. *, *p* < 0.05; **, *p* < 0.01.

In our study, kaempferol enhanced exercise performance and recovery by increasing the energy supply and antioxidant capacity and by decreasing the metabolite accumulation in the mice. However, the exact mechanism demands validation. Skeletal muscle is a key tissue for movement and metabolism in the body, and its precise regulation is crucial for maintaining body function. To evaluate the effect of kaempferol on glucose uptake, we treated the C2C12 myotubes with 2-NBDG, a fluorescently labeled glucose analog. The results (Figure 3A) demonstrated that compared with the control, the mean stain area of 2-NDBG was increased by kaempferol at higher concentrations (12.5, 25, and 50 μM (*p* < 0.05)). We next investigated the effect of kaempferol on protein synthesis, as determined using the SUnSET method. As demonstrated in Figure 3B, our in vitro study confirmed that kaempferol increased protein synthesis. The mitochondrial mass and mitochondria membrane potential—essential for maintaining muscle function and health—were also important effect indicators in our study. The stained areas of TMRM and MitoTracker were significantly larger in the 50 μm and 25 μm kaempferol treatment groups than in the control group (Figure 3C). To eliminate the potential artifact of increased mitochondrial mass on the membrane potential, the mitochondrial membrane potential was determined by the ratio of TMRM to MitoTracker. As shown in Figure 3C, the ratio of TMRM to MitoTracker was also significantly increased by 50 μm and 25 μm kaempferol. ROS is produced during exercise or energy production; excessive ROS during exercise can affect the normal function of cells and reduce muscle contraction and endurance. Kaempferol exhibited potent antioxidant properties, as evidenced by the decreased ROS levels (Figure 3D).

To elucidate whether the bioactivities of kaempferol were retained in a 3D microsphere, we examined its effects in C2C12 myotubes cultured in 3D (Figure 3E–H) and found that kaempferol significantly decreased the ROS in a dose-dependent manner in the C2C12 myotubes cultured in 3D. Moreover, kaempferol enhanced the fluorescence intensity of 2-NBDG, particularly at higher concentrations, compared with the control group, indicating that kaempferol pretreatment can increase glucose uptake. Similarly, kaempferol significantly increased the average fluorescence intensity of the puromycin, TMRM, and MitoTracker in the 3D microspheres; although there was an increasing trend in the ratio of TMRM to MitoTracker, it was not statistically significant compared to the control group (Figure 3H).

As the predominant fuel supporting muscle contraction, increased glucose uptake enables greater ATP generation through glycolysis and oxidative phosphorylation, thereby improving muscle strength and endurance capacity. Mitochondria are organelles in muscle cells responsible for energy production. Better mitochondrial mass means muscle cells can produce more ATP to provide the energy needed for muscle contraction, which is closely associated with muscle strength and exercise capacity [40]. Additionally, promoting protein synthesis is crucial for muscular hypertrophy and recovery from exercise-induced muscle damage [11]. To comprehensively investigate the effects of kaempferol on C2C12 cells, we used both conventional 2D cell culture systems and advanced 3D cell culture techniques to mimic the native microenvironment of cells in vivo. As the 3D culture more closely recapitulates the physiological milieu of cells in living organisms, these observations imply that the salutary effects of kaempferol on skeletal muscle cells observed in conventional 2D cultures are likely to translate to actual skeletal muscle tissues in vivo. In summary, kaempferol might facilitate muscular adaptation to exercise by stimulating protein synthesis, glucose uptake, and mitochondrial mass and by decreasing ROS. However, further in-depth research is required.

### 3.5. Effect of Kaempferol on MAPK and PI3K/AKT Signaling Pathways in C2C12 Myotubes

**Figure 4:**
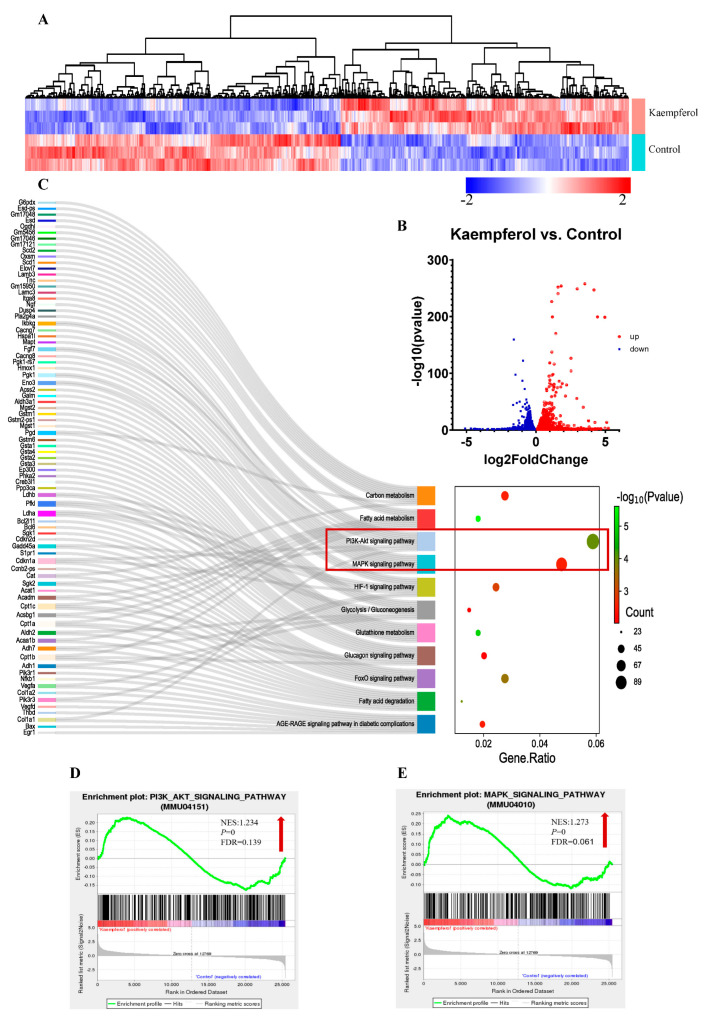
RNA-sequencing data induced by kaempferol in C2C12 myotubes. (**A**) Heatmap of gene expression levels in the control and kaempferol groups; (**B**) differentially expressed genes (DEGs) in C2C12 cells; *p* < 0.05 and |log_2_(Fold Change)| > 0 was seen as DEGs; (**C**) Sankey dot pathway enrichment (*p* < 0.05) from DEGs; and PI3K/AKT (**D**) and MAPK (**E**) signaling pathways of GSEA analysis.

To investigate the mechanism underlying the effect of kaempferol on exercise performance, we used transcriptome profiling to systemically identify changes in gene expression in C2C12 myotubes. Figure 4A manifests the diverse gene expression profiles in the three groups. We focused on DEGs, which exhibited being significantly upregulated (red dots, kaempferol vs. control) and downregulated (blue dots, kaempferol vs. control) in response to the kaempferol (Figure 4B). There were 2034 consistently upregulated genes and 2232 consistently downregulated genes. The bioinformatic analysis on the pathway enrichment of the DEGs indicated that kaempferol more significantly altered the MAPK and PI3K/AKT signaling pathways over other signaling pathways, as evident from the proximity of the Sankey dot enrichment pathway to the energy metabolism-related pathways (*p* < 0.05) (Figure 4C). The GSEA analysis of the MAPK and PI3K/AKT signaling pathways revealed that kaempferol significantly upregulated the MAPK (NES = 1.273, false discovery rate q-value, and FDR = 0.061, usually <0.25 is acceptable [41]) (Figure 4D) and PI3K/AKT (NES = 1.234 and FDR = 0.139) (Figure 4E) signaling pathways. The MAPK and PI3K/AKT signaling pathways regulate key processes in muscle, including growth, proliferation, differentiation, and metabolism [42,43]. Specifically, the MAPK signaling pathway mediates mitochondrial biogenesis, angiogenesis, and muscle hypertrophy, whereas PI3K/AKT affects glucose uptake, protein synthesis, and muscle regeneration [17,44].

### 3.6. Kaempferol Upregulated the Phosphorylation of Key Target Proteins in the MAPK and PI3K/AKT Signaling Pathways

**Figure 5:**
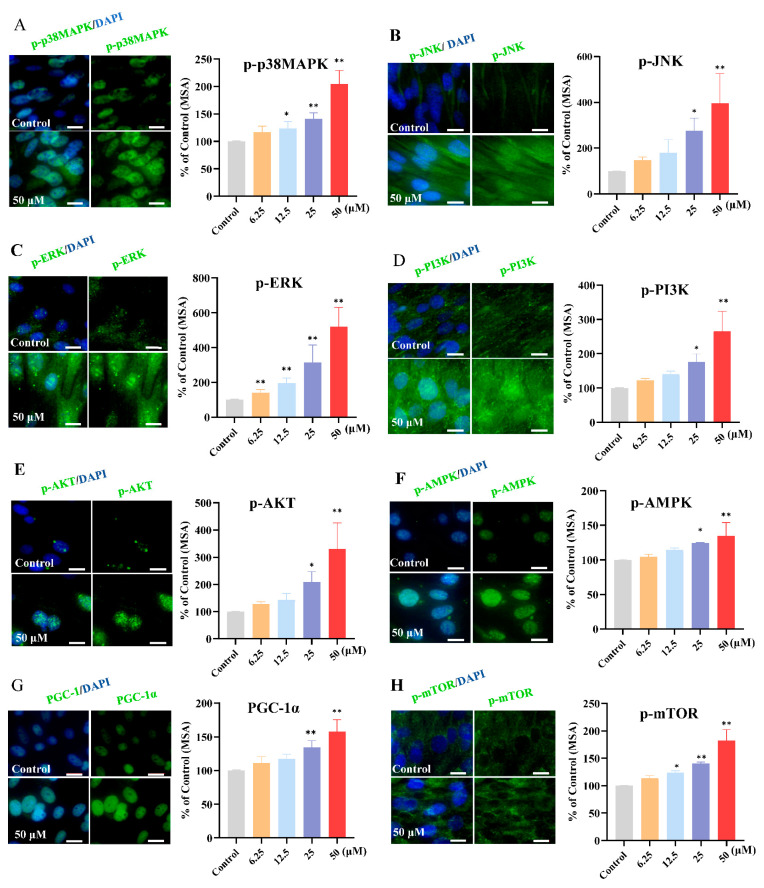
Protein expression/phosphorylation of markers of MAPK and PI3K-AKT signaling pathways. p-p38 MAPK (**A**), p-JNK (**B**), p-ERK (**C**), p-P13K (**D**), p-AKT (**E**), p-AMPK (**F**), PGC-1α (**G**), and mTOR (**H**) were detected with a high-content imaging analysis system. (**A**): representative images, bar size: 50 μm. The assays were performed in triplicate with three technical replicates. *, *p* < 0.05; **, *p* < 0.01.

To gain an insight into the mechanisms by which kaempferol affects muscle function in vitro, we first examined the phosphorylation expression of key target proteins in the PI3K/AKT (mainly including PI3K and AKT) and MAPK signaling pathways (p38 MAPK, JNK, and ERK). A 24 h exposure to kaempferol increased the phosphorylation levels of PI3K, AKT, p38 MAPK, JNK, and ERK in the C2C12 myotubes (Figure 5). Consistent with the MAPK and PI3K-AKT signaling pathways, studies have suggested that exercise fatigue-related abnormalities may be attributed to the dysregulation of the MAPK and PI3K-AKT signaling pathways [5,12,13]. Our findings suggest that kaempferol may upregulate the protein expression levels of the PI3K/AKT and MAPK signaling pathways to enhance exercise performance, consistent with the results of the transcriptomic analysis.

To further elucidate the multifaceted regulatory effects of kaempferol on glucose uptake, protein synthesis, and mitochondrial biogenesis in C2C12 cells, we examined the expression changes in AMPK, mTOR, and PGC-1α to investigate the molecular mechanisms by which kaempferol modulates muscle cell metabolism. Compared with the CT group, kaempferol incubation upregulated p-AMPK (1.25- and 1.35-fold by 25 and 50 μM kaempferol, respectively), PGC-1α (1.34- and 1.58-fold by 25 and 50 μM kaempferol, respectively), and p-mTOR (1.24-, 1.41-, and 1.82-fold by 12.5, 25, and 50 μM kaempferol, respectively) (Figure 5G,H). Our results demonstrated that kaempferol upregulated PGC-1α and phosphorylated AMPK and mTOR in C2C12 cells. As a critical regulator of energy metabolism, AMPK activation promotes ATP-generating pathways, including glucose uptake, mitochondrial biogenesis, and glycolysis [17]. Furthermore, studies have reported that AMPK might directly regulate PGC-1α, associated with the regulation of energy generation, modulating the expression of genes involved in fatty acid oxidation, oxidative phosphorylation, and mitochondrial biogenesis [10,45,46]. We speculated that the increased glucose uptake and mitochondrial biogenesis in C2C12 cells were likely because of kaempferol-induced AMPK activation. Moreover, mTOR is an indispensable component of the nutrient-sensing capability and overall coordination of cellular metabolism, and it plays a pivotal role in regulating protein synthesis and increasing muscle mass and strength [11]. Furthermore, complex crosstalk existed among those signaling pathways, which serve as an important mechanism of action for nutritional supplements. Specifically, the activation of the PI3K-AKT and MAPK signaling pathways can stimulate mTOR to promote protein synthesis and activate PGC-1α, which facilitates GLUT4 translocation and glucose uptake in muscle [11,13,47].

### 3.7. Effect of Kaempferol on Downstream Maker Related to Glucose Uptake, Protein Synthesis, and Mitochondrial Biogenesis

**Figure 6:**
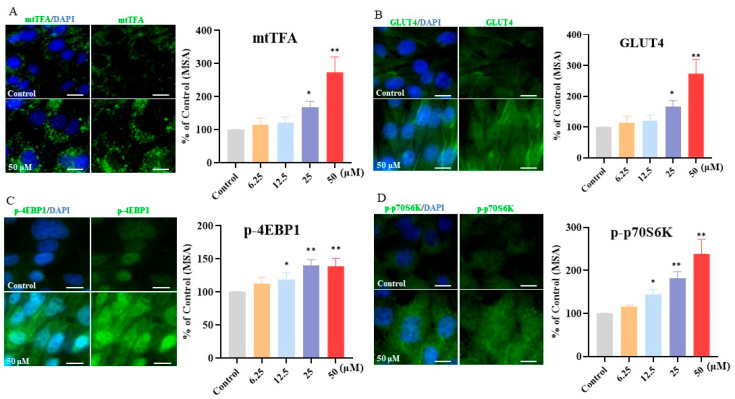
Protein expression/phosphorylation of downstream maker targeted to biological functions in C2C12 myotubes. Protein expression of mtTFA (**A**) and GLUT4 (**B**) and the phosphorylation of 4EBP1 (**C**) and p70S6K (**D**) were detected with a high-content imaging analysis system. Left panel: representative images, bar size: 50 μm. The assays were performed in triplicate with three technical replicates. *, *p* < 0.05; **, *p* < 0.01.

To better elucidate the mechanisms underlying kaempferol’s effects, we further focused on several key downstream targets closely associated with the observed bioactivities. As presented in Figure 6, compared with the CT group, kaempferol upregulated mtTFA (*p* < 0.05 in 25 μM kaempferol and *p* < 0.01 in 50 μM kaempferol) and GLUT4 (*p* < 0.05 in 25 μM kaempferol and *p* < 0.01 in 50 μM kaempferol); as well, kaempferol induced a dose-dependent increase in the phosphorylation of 4EBP1 (*p* < 0.05 in 12.5 μM kaempferol, and *p* < 0.01 in 25 and 50 μM kaempferol) and p70S6K (*p* < 0.05 in 12.5 μM kaempferol, and *p* < 0.01 in 25 and 50 μM kaempferol). Kaempferol upregulated mtTFA and GLUT4 in a dose-dependent manner, with enhanced phosphorylation of 4EBP1 and p70S6K.

Our results demonstrated that kaempferol upregulated mtTFA, a key downstream target of activated PGC-1α. PGC-1α is a point of convergence for diverse signals that stimulate mitochondrial biogenesis. Activated PGC-1α interacts with transcription factors, including nuclear respiratory factor 1 (NRF1) and nuclear respiratory factor 2 (NRF2), to induce genes involved in mitochondrial biogenesis, thereby stimulating the expression of mtTFA—a major regulator of mitochondrial DNA transcription and replication [48,49]. Therefore, the kaempferol-mediated upregulation of mtTFA observed here likely occurred through the upstream activation of the PGC-1α. Additionally, the activation of PGC-1α might result in the stimulation of GLUT4. Increased GLUT4 expression, critical for skeletal muscle glucose uptake and maintaining whole-body glucose homeostasis, probably facilitated greater glucose uptake [43]. 4EBP1 and p70S6K were key effectors regulating mRNA translation and protein synthesis downstream of mTOR; their activation by kaempferol thus provided a mechanistic basis for the increased protein synthesis observed. In summary, kaempferol enhances exercise performance by upregulating phosphorylation of the MAPK and PI3K/AKT signaling pathways and coordinately activating the critical metabolic regulators—AMPK, mTOR, and PGC-1α—in cells.

In our study, we found that kaempferol could significantly improve exercise performance and we have preliminarily proposed the mechanisms which provided a good basis for subsequent research. The in vivo study’s findings do not indicate toxicity of kaempferol but rather highlight its positive effects on human health, such as anti-inflammatory, anticancer, antioxidant, and antidiabetic properties [25]. However, the safety concerns regarding kaempferol, especially the relationship between intake levels and health benefits, will be a key focus of future research. This study still has some limitations. Firstly, the stability of kaempferol in the culture medium over 24~48 h or at different time points is also an important issue worth investigating. The specific pathways/effectors involved in this study are conducted in cells, and further in-depth research in animal models and even human populations will be necessary in the future. The next issue is the safety of kaempferol. In our preliminary experiments, we conducted an acute experiment with a maximum gastric gavage dose of 5 g/kg bw in mice and observed no mortality. However, this safety study was insufficient. A more comprehensive safety assessment is still needed. Currently, we have verified the bioactive effects of kaempferol in cell and animal experiments; the specific pathways/effectors involved in this study especially were only conducted in cells. In future studies, it will be crucial to translate the obtained results to the human population. Specifically, clinical or human physiological experiments are necessary to investigate the impacts and efficacy of kaempferol in individuals.

## 4. Conclusions

**Figure 7:**
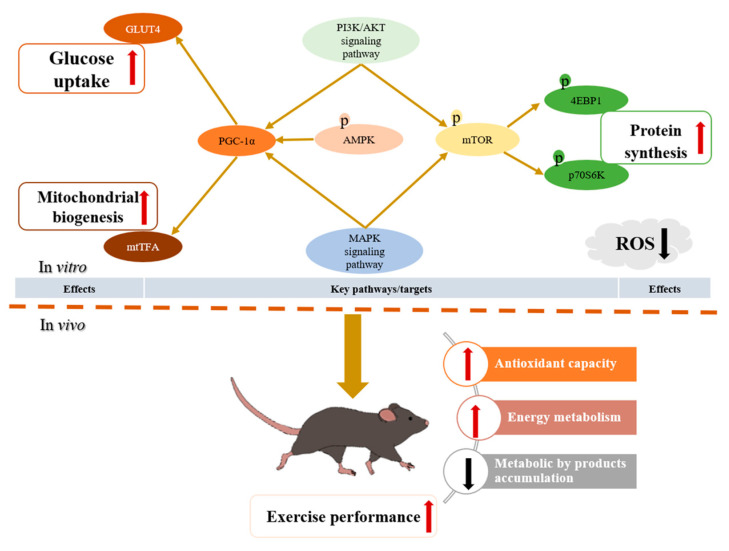
Summary of the effects and mechanism of kaempferol to enhance exercise performance.

In summary, kaempferol is a natural bioactive component in various foods and herbal medicines, where it demonstrates antioxidant and anti-inflammatory effects. As an advantageous active component, the main benefits and potential mechanisms by which kaempferol improves exercise performance are diagrammatically represented in Figure 7. Kaempferol effectively enhances exercise performance by improving grip strength, endurance duration, and distance while also enhancing antioxidant capability, energy metabolism, and decreasing metabolic byproducts during exercise. Furthermore, in vitro studies conducted on C2C12 muscle cells revealed the following: kaempferol reduced ROS generation, which not only minimized ROS damage to C2C12 but also demonstrated a fundamental cellular protection, aiding in regulation of other biological processes; kaempferol activated the PI3K/AKT and MAPK signaling pathways, enhancing biological functions; kaempferol stimulated AMPK and PGC1-1α, inducing GLUT4 expression for enhanced glucose uptake while simultaneously promoting mtTFA and mitochondrial transcription/translation, elevating mitochondrial biogenesis; and concurrently, kaempferol upregulated mTOR, leading to downstream protein synthesis by the critical targets 4EBP1 and p70S6K.

## Data Availability

The original contributions presented in this study are included in the article/Supplementary Material; further inquiries can be directed to the corresponding author.

## Supplementary Materials

The following supporting information can be downloaded at: https://www.mdpi.com/article/10.3390/foods13071068/s1, Figure S1: Effect of kaempferol on serum biochemical parameters in mice. (A), aspartate aminotransferase (ALT); (B), alanine aminotransferase (ALT); CT, Control group, 0.5% (wt./vol) solvent carboxymethyl cellulose sodium (CMC); KL, 25 mg/kg bw of kaempferol; KH, 100 mg/kg bw of kaempferol; bw, Body weight. *, *p* < 0.05; **, *p* < 0.01.

## Abbreviations

2-NBDG: 2-[N-7-nitrobenz-2-oxa-1,3-diazol-4-yl)amino]-2-deoxy-Dglucose; 4EBP1, 4E binding protein-1; ALT, alanine aminotransferase; ANOVA, analysis of variants; AST, aspartate aminotransferase; ATP, adenosine triphosphate; BUN, blood urea nitrogen; CK, creatine kinase; CMC, carboxymethyl cellulose sodium; DEGs, differentially expressed genes; DMEM, Dulbecco’s Modified Eagle’s Medium; FBS, fetal bovine serum; GLUT4, glucose transporter-4; GSEA, gene set enrichment analysis; GSH-Px, glutathione peroxidase; KEGG, Kyoto Encyclopedia of Genes and Genomes; LA, lactic acid; LDH, lactate dehydrogenase; LSD, least significant difference; MDA, malondialdehyde; mTOR, mammalian target of rapamycin; mtTFA, mitochondrial transcription factor A; NCBI–GEO, National Center for Biotechnology Information Gene Expression Omnibus; NRF1, nuclear respiratory factor 1; NRF2, nuclear respiratory factor 2; P/S, penicillin-streptomycin; p70S6K, ribosomal protein S6 kinase; PBS, phosphate-buffered saline; PGC-1α, peroxisome proliferator-activated receptor gamma coactivator 1-alpha; ROS, reactive oxygen species; SD, standard deviation; SOD, superoxide dismutase; TBHP, tert-butyl hydroperoxide; TC, total cholesterol; TG, triglyceride.
