# Supplementary material for: Kaempferol Improves Exercise Performance by Regulating Glucose Uptake, Mitochondrial Biogenesis, and Protein Synthesis via PI3K/AKT and MAPK Signaling Pathways
Source: Foods. 2024 Mar 30;13(7):1068. doi: 10.3390/foods13071068 (PMC11011654; doi:10.3390/foods13071068)
Supplement: Supplementary file 1 [file foods-13-01068-s001.zip › foods-2913482-supplementary.pdf]

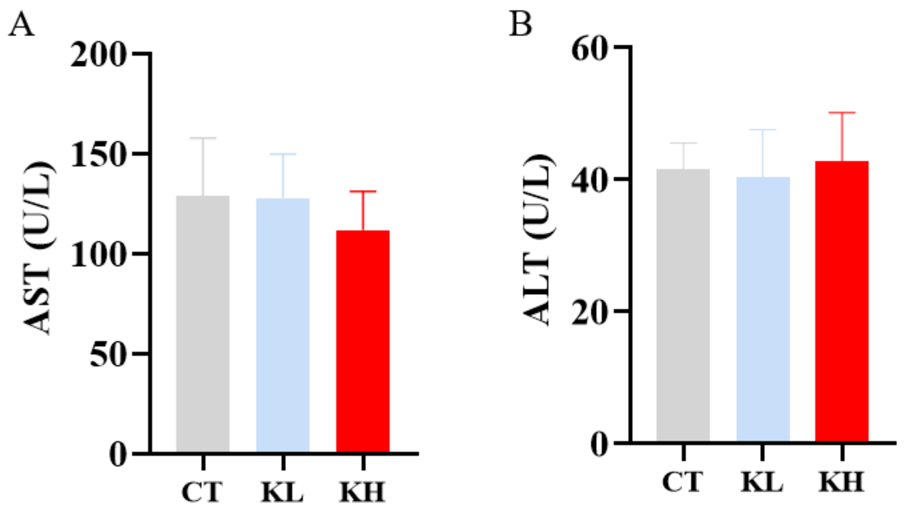

Figure S1. Effect of kaempferol on serum biochemical parameters in mice. Figure S1: Effect of kaempferol on serum biochemical parameters in mice. (A), aspartate aminotransferase (ALT); (B), alanine aminotransferase (ALT); CT, Control group, 0.5% (wt./vol) sol-vent carboxymethyl cellulose sodium (CMC); KL, 25 mg/kg bw of kaempferol; KH, 100 mg/kg bw of kaempferol; bw, Body weight. *, $p < 0.05$; **, $p < 0.01$.
